# The Progressive Development of Physiological Ideas

**Published:** 1858-01

**Authors:** 


### *The Progressive Development of Physiological Ideas.*

Read before the Detroit Medical Society by E. P. Christian, M. D., and published by request.

The birth of ideas originating the discovery of new laws in the economy of nature, and of their application in explaining and interpreting the various and manifold natural phenomena, or the application of natural forces, science and the arts, is not like that of the fabled goddess Minerva, springing full formed and of perfect development into life from the head of Jove, except in the manner expressed in this alligory, representing wisdom emanating from the head of the Supreme Governor and Ruler of the Universe—the Omniscient Divinity from whom all knowledge proceeds.

Occasionally the world furnishes examples of intellects of that acuteness and capacity for observation and ingenious deduction, who seize upon the embryonic ideas of their predecessors, and bring forth such admirable order and system from chaotic confusion that the advances they make seem like the discovery and complete development of new and unheard of laws.

The idea of the application of steam as a motive force underwent a gestation of ages before its development and birth into the steam engine; and so too the electric telegraph was the product of the gradual growth of ideas concived e'er yet Franklin received his message from the clouds.

This fact of the progressive development of great ideas is sufficiently exemplified by what is of common observation, that new discoveries are seldom without competing claimants for the credit of priority.

The study of the history of the development of those ideas which have led to the elucidation of new principles, the explanation of mysterious phenomena or new application in the arts, will prove both an interesting and profitable pursuit. By this means only shall we be capable of judging of the boasted progress of successive ages over the preceding, and in particular of the vain-glorious claims of our own age to pre-eminence over all that has preceded. And so too shall we be enabled to render due credit to the fameworthy labors of older writers, as we shall learn that what appears but the pale flickering light sending dimly back its rays is only rendered so by its distance from the beholder, or perhaps by the denseness of the surrounding fog through which the glimmering of its rays is seen.

Young Physic, a zealous student of modern medical literaure, both in its teeming periodicals anu the more ponderous volumes daily emanating from the press, and familiar with modern discoveries and improvements, is inclined to irreverence for what presents the mustiness of age; but an equal familiarity with more ancient literature would often show him how the fire which burns so brilliantly for him, was lighted years before, and its flames kept shining by continual addition of fuel by successive watchers.

Old Fogy Physic too, remembering the proud advances in science of his younger days, not seldom permits his inertia the satisfaction of a neglect of study of more recent writings, and thus perhaps fails to see by what process the flickering taper of former years has gradually given place to the brilliant carbo-hydrogen illuminators.

Each discovery is the result of the progressive developments of some principle, and is dependent upon preceding developments of the same; and so too, each discovery opens new indications to still farther truths.

In no department of science has this truth been more distinctly shown than in physiology, and in nothing pertaining to the human body is it more impressively manifested, "how fearfully and wonderfully made is man," than in the physiology of the nervous system. What wonderful mechanism and design is there displayed, and what startling, mysterious phenomena, that were wont of old to be charged to demoniac influence, have been shown to depend upon the influence of this system.

But marvelous as have been the developments of science here, still greater mysteries will yet be explained, and future discoveries will prove that our age was but the infancy of nervous physiology.

Here, however, on this dark, uncertain subject we are to bear in mind especially, that hypothesis merely, howsoever specious, is not discovery, if it lack demonstrative proof. Credit nevertheless is due to those whose foresight has enabled them to perceive what has remained for more fortunate ones to demonstrate; but how often is it that theories have been broached as new, and great discoveries heralded, when a more familiar acquaintance with medical literature would have shown the same to have been conceived and given birth to by others. Not often, however, is it that competing claimants arise for the credit of such so called discoveries, and when such is the case, that numerous contestants enter the ring, this fact is of itself sufficient to prove the fallacy of any exclusive claim, and to indicate that the idea was a common one.

Such a contest is now exhibited by the parties claiming priority in the discovery and designation of what is styled *excito secretory system of nerves.*

Our design in this paper is to trace up the development of this asserted new discovery, and to show what these rampant partisans are entitled to, and how much has been borrowed from older authors on the physiology of the nervous system. For this purpose a clear understanding of the nature of these claims will be necessary.

Dr. H. F. Campbell, of Georgia, claims the idea and designation of an excito-secretory action, prior to Dr. Marshall Hall. This claim is acknowledged valid by Dr. M. Hall, who however, awards the credit of antecedent demonstration of facts in proof of this doctrine to M. Bernard. Dr. J. Adams Allen, of Michigan, contests the claim to priority of discovery with Dr. Campbell, and Dr. M. Paine, of New York, asserts vigorously his claims to having anticipated each and all the others.

We may perhaps better show the nature of the contest by quotiug the claims of the interested parties.

"Dr. Campbell," says Marshall Hall, "is entitled to the idea and designation of an excito-secretory action, but his details are limited to pathology and observation."

That "the idea of the excito-secretory function, as applied to pathology, is indisputably his; he first detected, gave it its designation and saw its vast importance."

"My own claim," says he, "consists in the vast generalization of excito-secretory action throughout the system."

"The elaborate experimental demonstration of reflex-excito-secretory action is the result of the experimental labors of M. Claude Bernard."

Dr. Allen claims "the great generalization that the excito-influence is followed by a reflex change, in which the effect is not a motion, but a modification of vascular and nutrient action. That this effect takes place by means of the double nervous arc.'"

He remarks, "as Mr. Hall is kind enough to allow Dr. Campbell the credit of having discovered the excito-secretory function, "as applied to pathology," perhaps he may be led to recognize my own claim to the discovery of this principle, as applied to therapeutics. I shall not be content with this," &c.

He then quotes from his old manuscript lecture which is adduced in proof of the priority of his discovery, as follows:

"Hence the impression must be transmitted to the nervous centres, and thence reflected to the affected organ; in other words, the influence is primarily exerted upon the cerebro-spinal system, and secondarily upon the internal affected organ."

Dr. Paine claims, that the whole of this doctrine contained in the first extract from Dr. Allen "is impressed upon the medical and physiological commentaries, (of which he was the author,) and upon half the pages of these Institutes (of Medicine), and has always been taught extensively in the Author's lectures since 1841."

And again of the second quotation from Dr. Allen, in regard to the application of the principle to therapeutics and his explanation of the modus operandi of medicines, Dr. Paine remaks as follows:

"Here also the whole of the foregoing doctrine appears throughout these Institutes, but they embrace a long chapter particularly upon "counter-irritation," in which it will be seen that the author has employed nearly the foregoing language of Dr. Allen and with a great elaboration and extensive application of the doctrine throughout the work, which had been also antecedently taught in his lectures for seven consecutive years before Dr. Allen promulgated his views. He enforces everywhere the doctrine that reflex action of the nervous power is the modifying cause through which all changes are effected by morbific and remedial agent in parts that are not immediately connected with the direct seat of their action," &c.

And again says Dr. Paine: "As to M. Bernard, his experiments bearing upon the connection of the nerves with the function of secretion, however much they may have been varied and multiplied, were anticipated long before by those of A. W. P. Phillip, which are quoted extensively in these Institutes."

Here then is an amusing contention; and what does it indicate, but, as has been before said, that the principle laid claim to by these contestants has been no hidden thing—may be traced as far back as the experiments of Phillip above referred to? This is, indeed, the whole discovery. There has been no new system discovered. No one professes to point out the newest fibre, ramification or ganglion. It is then merely the discovery of a function, and these modern claimants do not even point out the channel of this communication. They do not tell us what system is the medium of this newly discovered force. What then does this mean? If they cannot tell us by what system this function is performed, and give us some plausible demonstration, what does this whole theory amount to but baseles, hypothesis? But if they leave us to infer, as we must, that the function is performed through the medium of the nerves of organic life, do they not do so because it is an admitted fact in physiology of the presidence of the great sympathetic over the organic viscera; and where then is the discovery? We have admitted such a function to this nerve before. Dr. Campbell, indeed we may infer from the tenor of his very excellent report on the nature of Typhoid Fevers, in the Vol. of Transaction of the American Medical Association for 1853, places the seat of this function in this system of nerves.

This idea then we have claimed is as old as the experiments of Phillip, and we shall attempt to show that it may be definitely traced up to Bichat, and may be found more distinctly enunciated by Broussais, and unequivocally so by subsequent writers. Probably there is not a gray haired man in the profession, but who recognizes in these ideas the familiar doctrines of older writers; and within the writer's own knowledge the elaboration of this very same principle, as applied to the pathology of malarious fever, was contained in a thesis by one of our own members, more than a score of years since. Indeed, the very idea is involved in the terms made use of by the older writers, and which have also been copied by modern ones, as "sympathy," and the "great sympathetic," and "nerves of organic life."

Let us see then, if we can furnish any evidence to justify us in these statements.

Even as old a writer as Bichat had at least a faint perception of this truth; and the germ of the whole theory is developed in his attempt to prove that "the passions regulate the actions of animal life though they have their seat in organic life." Imbued, as he was, with the materialistic ideas of French philosophy which prevailed in the days of the Republic, he seeks to reconcile his physiology with his moral philosophy. Attempting to separate the organic functions from the direct nervous influence of the brain, he regards each ganglion a separate and independent nervous centre, presiding over those organs to which its emerging fibres are distributed. And observing the influence of the passions and emotions on these functions, he attempts to prove from thence their seat and origin in these centres; yet he is necessitated to acknowledge, that "numerous sympathetic relations unite all the internal viscera with the brain or its different parts. Every day's experience (says he) affords us examples of affections of this organ arising sympatheticallly from those of the stomach, liver, intestines, spleen, &c. This being premised, as the effect of every kind of passion is to produce an affection, a change of force, in some one of these viscera, it will also excite sympathetically either the brain wholly, or only some of its parts, whose reaction on the muscles which receive it from the nerves, will produce in them those motions which we observe."—*Phys. Researches on Life and Death, pub.* 1809, *p.* 54.

Here, then, we have unequivocally the theory of excito-motory and reflex action, which, as we shall see, is the parent of this theory.

But again, says he, "Perhaps the internal organs do not act upon the voluntary muscles by the intermediate excitement of the brain, but by direct nervous communications; how they act is of no consequence, * * what is most essential, is the fact itself; what is evidently in support of it, is, on the one part, the affection of an internal organ by the passions; on the other part the determinate motion to this affection in muscles, over which this organ has no influence in the ordinary series of phenomena," &c.—*Ibid.*, *p.* 55.

"It is from the affection, and not from the cause which produces it that sympathy arises."—p. 55.

It is not only in the brain, but on every other part, that the viscera affected by the passions exercise their sympathetic influence," *&c.*—p. 56.

Thus much then, from Bichat, one of the brightest lights of medical literature, who was prevented only by his false metaphysics, in placing the origin of the passions, emotions *&c.*, in the sympathetic ganglions, the necessary result of his peculiar infidelity, from anticipating the glory of succeeding physiologists.

We will next refer to the writings of Broussais, and select the following extracts:

"It is proved that the nerves coming from the brain are the channel for the sensations, which, from various parts of the body, go to the center of perception and volition; that is to say, the influences under which the latter determines movements. These two phenomena which constitute *innervation* are, on final analysis, but modes of general irritation.

The cords of the great sympathetic are continuous with the cerebral nerves, and are to be considered like these latter, as conductors of irritation. This granted, it must, of necessity, follow that irritations which are developed in the viscera, where the great sympathetic predominates, should be communicated to the cerebral nerves, and by them conducted to the encephalic center.

It is also, equally indispensable to admit that the irritations of volitions emanating from the brain should be carried into the ganglionic nerves, and penetrate by means of these latter into the tissues in which these nerves are distributed.

There is then, reciprocity of stimulation between the encephalic and ganglionic nerves; that is to say they serve as excitors of each other."—*Broussais' Physiology*, 1832, *p.* 261.

Again, " Sometimes excessive pains, even of external parts to which the great sympathetic does not extend, such as of the skin, and the articulations, determines an involuntary agitation, and may even go so far as to excite delirium, as I have seen in a most violent case of arthritis."

"To this I would reply that, all the perceptions being as I have proved, reflected from the brain to the viscera, the irritation caused by an excessive pain must be transmitted to the great sympathetic," &c., &c.—*Ibid*, *p*. 262.

"The cords of this nerve which plunge into the large secretors annexed to the hollow organs, as the salivary gland the pancreas and the liver, must associate the secretions on the one side, with the mucous membrane of the alimentary canal, and on the other, with the brain," &c., &c.— *Ibid. p.* 268.

"The encephalic nerves establish relations with external bodies, and preside over the grander movements, as those of the muscular masses for locomotion.

"The great sympathetic establishes in the interior of the body, relations between the viscera, and regulates their pasricular movements. For the exercise of this function, it borrows stimulation from the encephalon, and transmits some to it at need"

"The great sympathetic receives stimulation from the cerebral nerves, which are themselves indebted for it to the action of external bodies, and make use of it to bring into play the cephalo-splanchnic and splanchnic muscles, and the coats of the arteries," &c., &c.— *Ibid, p.* 272.

Mark that! Do not the above extracts, and particularly the last two contain a very clear enunciation of not only the excito-motery, but also of the excito-secretory function; and this too, even before the full elaborate demonstrative experiments of Sir Charles Bell, which established as an admitted truth the fact of the existence of the two sets of nerves in the spinal column. But even in this we are anticipating. The work in which these doctrines are enunciated was given to the world about 1832.—Bichat's work had preceded by a score of years—and now by going back, even anterior to Broussais, we shall find clear expressions given to the idea involved in this theory. Thus the following extract from Pereira in regard to the experiments of Sir Benj. Brodie, published in 1811–12.

"It has been generally supposed that there were two modes by which medicines or poisons affected remote parts; these were absorption or the passage of medicinal or poisonous molcules into the blood, and by sympathy, or by an impression transmitted through the nerves."—*Pereira, Mat. Med.*, v 1, *p.* 148.

And says the same author, Messrs. Morgan and Addison advocated the operation of medicines by sympathy as early as 1849.

It was about the same time from 1834 to 1836, that Sir Charles Bell was engaged on his demonstration of the double set of nerves, the sensitive and motor, and only two years subsequent to the last date, in 1838, that Marshall Hall announced his excito-motor theory; naturally arising from the demonstrations of Sir Charles Bell, and which in fact we may regard as a modification, or perhaps an elaboration of it, as no other system of nerves is demonstrated; but the function only. For of the existence of other sets of nerves distinct from those of Sir Charles Bell, there is no positive anatomical proof, the only proof, consisting in the fact that, on the hypothesis of the existence of this system of nerves could otherwise inexplicable physiological phenomena be plausibly explained. And in truth, this was a plausible hypothesis bearing at one time almost the force of demonstration. But at this time other hypotheses are advanced furnishing an equally plausible explanation of these phenomena—thus, the hypothesis being adopted by Todd and Bowman, and which indeed, appears to be the most simple and philosophical explanation, "that the mechanism of a mental and physical nervous action are essentially the same, differing only in the nature and mode of application of the stimulus. The same afferent and efferent fibres are exerted in the one case as in the other; the former acting as sensitive or excitor, or both; and the latter as channels for *voluntary emotional*, or strictly physical impulses to motion."

Such was the discovery of Marshall Hall, heralded as the great discovery of the age, which has immortalized his name, like others of his leading ideas, simply by the noise in proclaiming them, and the zeal and steadfastness of purpose in making them popular, and urging their importance.

Again we will quote from Pereira; "The agents whose operation is of the kind here referred to, (electricity, heat, cold, light, mechanical irritants, corrosives, &c.,) affect remote parts by the agency of the true spinal aud ganglionic system. The mode of action of those which act through the true spinal system is excited and reflex, that is, an impression is made on and caried by the incident excitor nerves to the nervous center, which, by its peculiar power, affects a remote part through the medium of its reflex motor nerves."

"The mode of operation of those agents which act through the ganglionic system is excited, and may, perhaps, also be reflex.—*Pereira, a.* 1. *p.* 163.

If this be not an explanation of the excito-secretory theory, applied to therapeutics, we greatly misapprehend what is claimed.

We have thus traced up the development and modifications of his idea, from the time of Bichat We would not detract from the merit which attaches to each of these modern claimants for their elaboration and application of the truth, each in his own separate way—but we do claim that the merit of originality in the advocating the hypothesis, or in practical demonstration of the principle, can be exclusively yielded to none of them, and that a just adjudication would restore the glory to older authors, which is in danger of being snatched from them.—*Peninsular Journal of Medicine.*

